# High-Quality Black Phosphorus Quantum Dots Fabricated via Microwave-Tailored Technology

**DOI:** 10.3390/nano10010139

**Published:** 2020-01-13

**Authors:** Kaixiang Du, Wen Yang, Shukang Deng, Xueming Li, Peizhi Yang

**Affiliations:** Key Laboratory of Renewable Energy Advanced Materials and Manufacturing Technology, Ministry of Education, Yunnan Normal University, Kunming 650500, Yunnan, Chinawenyang1972@hotmail.com (W.Y.); Skdeng@126.com (S.D.); lxmscience@163.com (X.L.)

**Keywords:** 2D materials, black phosphorus quantum dots, mineralization route, microwave-assisted technology, crystal disintegration

## Abstract

Black phosphorus quantum dots (BPQDs) have recently obtained great attention due to their high mobility and tunable bandgap features, which are beneficial for their potential application in photoelectronic devices. However, a precise synthesis of high-quality BPQDs is still a great challenge owing to the formation of an impurity phase when employing traditional methods. Herein, we demonstrate the scalable fabrication of BPQDs from mineralization-derived bulk black phosphorus (BP) single crystals by means of a microwave (MW)-assisted liquid-phase exfoliation method in ethanol. The primary results demonstrate that ethanol plays a crucial role in determining the final properties of BPQDs, such as their excellent tolerance to oxygen, good crystallinity, and uniform size. Furthermore, the mechanism behind the formation of BPQDs is proposed, revealing that a layer-by-layer disintegration process of bulk BP crystals under microwave-energy stimuli is responsible. This work may provide a novel path for the further development of BPQDs and corresponding devices.

## 1. Introduction

Two-dimensional (2D) layered materials, such as representative graphene [1,2], black phosphorus (BP), and transitional metal dichalcogenides (TMDs), have shown great potential application in the fields of electronics and optoelectronics [3,4], which is mainly due to their distinguished structural and electronic characteristics. Until now, state-of-the-art lower-dimensional materials, including 2D or 0D (quantum dots), have generally been exfoliated from bulk materials via chemical or physical methods, resulting in dramatically changed electronic and optical properties compared to those of the bulk materials [5,6,7]. Given the advantages of liquid-phase exfoliation (LPE) technology, such as its scalability, solution processability, and feasibility for creating composite materials [8,9], recently, several groups have produced black phosphorus quantum dots (BPQDs) in anhydrous organic solvents [10,11,12]. However, these solvents are toxic or have high boiling points that are detrimental to large-scale production, and they are difficult to remove upon assembly into a film or composite, especially for BPQDs, which have inferior stability. As a result, the precise analysis of their physicochemical properties and their utilization in micro–nano electronic devices is inevitably limited. Furthermore, the tedious processing time (for example, over 5 h) decreases the crystallinity and creates structural defects [13,14]. Therefore, the development of a novel, environmentally friendly strategy to fabricate BPQDs is urgently needed.

As is well known, the quality of BPQDs is highly dependent on the bulk material’s structure when the top-to-bottom strategy is employed to fabricate high-quality BPQDs. When considering a minimal number of defects, the bulk BP single crystal may be the best choice. Unfortunately, it is still a technological challenge to achieve this goal although several routes have been developed, such as the high-pressure method [15], the bismuth-flux routes [16], and mechanical milling technology [17]. These methods either use toxic chemicals or complex apparatus, are time consuming, or only provide small BP crystals or BP nanoparticles [18]. Herein, we demonstrate high-quality BP single crystals grown via the mineralization route. Compared to the above-mentioned methods, the mineralization route shows superior advantages for the efficient synthesis of high-quality BP single crystals under simple and gentle conditions [19,20,21]. On the basis of these high-quality BP single crystals, an efficient and fast technology to produce excellent quality BPQDs via microwave (MW)-assisted LPE technology in ethanol solution is also reported in this work. According to careful characterization, BPQDs are well crystallized with an average size of 2.4 ± 0.85 nm and a thickness of 2.19 ± 1.33 nm. It should be noted that this technology features a non-toxic ethanol solution and a short processing time. More importantly, BPQD samples are free of impurities and demonstrate excellent long-term stability in the air, which is beneficial for retaining the high quality of their bulk crystals for further analysis and application.

## 2. Materials and Methods

### 2.1. Growth of High-Quality BP Single Crystals

Red phosphorus lump (RP, 400 mg, 99.999%), iodine crystalline (I_2_, 20 mg, 99.99%), and tin shot (Sn, 30 mg, 99.999%) were sealed in an evacuated silica glass ampoule (11 cm in length and 10 mm in diameter). The ampoule was horizontally placed into a tube furnace, and the temperature was slowly increased to 620 °C. Subsequently, after being kept at 620 °C for 3 h, the temperature was decreased to 500 °C at a rate of 20 °C h^−1^. After that, the setting temperature was kept at 500 °C for at least 30 min and followed with a natural cooling process. We obtained an almost full conversion of RP to BP single crystals by prolonging the reaction time. Finally, the BP samples, grown as described above, were picked out and washed with ethanol several times to remove the residual mineralizer. The BP crystals were kept in a glovebox for further analysis. 

### 2.2. Preparation of BPQDs

The bulk BP was ground to BP powder, and then the BP powder (25 mg) was immersed in 5 mL of ethanol. Subsequently, the mixture was treated with the first MW system (MW-1) for 11 min at 70 °C, followed by being kept for 30 min at 80 °C using the second MW system (MW-2). After that, the solution was centrifuged at 6000 rpm for 30 min to remove any non-exfoliated BP powder. Finally, the top 50% of the supernatant was collected for further analysis and application.

## 3. Results and Discussion

### 3.1. Synthesis of High-Quality BP Single Crystals

**Figure 1:**
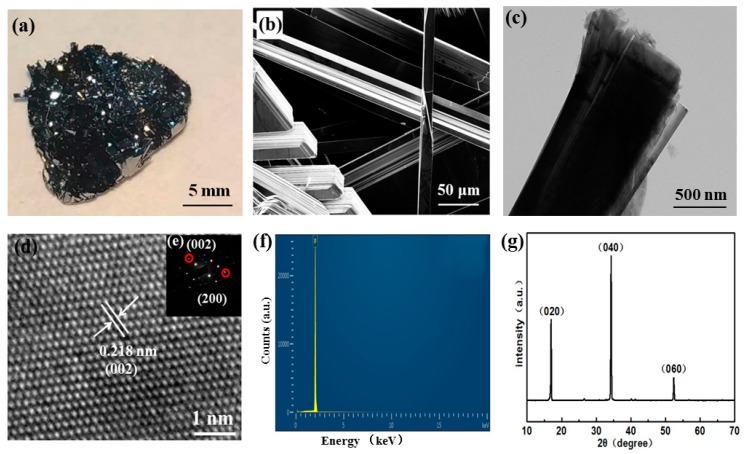
Structure and properties of black phosphorus (BP) crystals grown by the mineralization method: (**a**) photograph of a 1.5 cm BP single crystal; (**b**) SEM image and (**c**) low-magnification and (**d**) high-resolution TEM images of the BP crystal; and (**e**) selected area electron diffraction (SAED) pattern, (**f**) EDS spectrum, and (**g**) XRD pattern of the BP single crystal.

The large-sized BP single crystals were synthesized through temperature-programmed reactions, including a high-temperature platform for several hours and a slow cooling process, the conditions of which can be found in the experimental section. As seen in Figure 1a, a 1.5 cm bulk BP polycrystal with metallic luster was obtained after the reaction. To better understand the morphology of BP single crystals prepared in this way, a SEM image of the corresponding BP single crystals (see Figure 1b) was produced, which indicated that these BP crystals were uniform in thickness with micro-ribbon morphology. The layered structure, which can be cross-checked from the side view, provides the precondition to obtaining high-quality BPQDs by means of microwave (MW)-assisted LPE technology in ethanol solution. Subsequently, the morphology and crystalline structure of the bulk BP was further characterized using a transmission electron microscope (TEM) image. As shown in Figure 1c,d, a ribbon-like structure was clearly observed with a clear lattice spacing of 0.218 nm, corresponding to the (002) planes of BP crystals, which is good in relation to previously reported values of BP single crystals [21,22,23]. To further confirm the characteristics of the BP crystals, selected area electron diffraction (SAED) was conducted (Figure 1e, upper right inset of Figure 1d), which demonstrated the excellent crystallization of the corresponding materials. As reported previously, both high purity and good crystallinity are extremely important for keeping BP stable in air [24,25,26].

Aiming to reveal the components, the EDS spectrum of the prepared BP crystals is given in Figure 1f. Obviously, only the P element was detected, without any other element. This is an indicator of the formation of high-purity BP materials. Furthermore, the XRD pattern of the bulk BP crystalline, presented in Figure 1g, revealed that the preferred growth crystallographic planes were all (0k0) planes in which the peaks were mainly located at ~16°, 34°, and 52°, corresponding to characteristic diffraction peaks of (020), (040), and (060) planes of orthorhombic BP (space group Cmca (no. 64)) [21,22,23]. Therefore, it can be concluded that BP single crystals were successfully fabricated using this mineralization route, endowing the possibility of precise analysis and application without purification in the future.

During the temperature-programmed process, iodine and tin first evaporate to form SnI_4_ and SnI_2_. After the temperature reaches higher than 340 °C, SnI_4_ decomposes into SnI_2_. When the temperature rises to about 500 °C, red phosphorus starts to sublimate and form gaseous P, and when it continues to rise to 620 °C, RP completely transforms into gaseous P. Since Sn has a melting point of 231.9 °C and a boiling point of 2602 °C, most of the Sn is in liquid state, and a small part of it forms gaseous Sn. The reactions can be summarized as follows:

I_2 (S)_ → I_2 (G)_ (1)

Sn _(S)_ → Sn _(G)_ + Sn _(L)_ (2)

Sn + I_2_ → SnI_4_ Sn + I_2_ → SnI_2_ (3)

SnI_4_ → SnI_2_ + I_2_ (4)

RP → P_4 (G)_ (5)

where S is the solid state, L is the liquid state, and G is the gas state. In the subsequent cooling process, SnI_2_, Sn, and P_4_ react to form Sn_24_P_19.3_I_8_ [27,28]. A small amount of gaseous P inevitably forms Hittorf’s phosphorus (HP), deposited on the bottom of the quartz tube [27]. Subsequently, when the temperature is lowered to about 500 °C, the HP acts as a heterogeneous nucleation site for BP nucleation and growth. When the P_4_ molecule is exhausted, the reaction ends. However, at this stage, the time required for black phosphorus to grow is relatively short, and phosphorus can be completely converted to black phosphorus in 10 h. A temperature of 500 °C is critical for BP growth, as it properly prolongs its holding time and helps single crystal BP growth. The cooling process can be summarized by the following equations:

SnI_2_ +Sn + P_4_ ↔ Sn_24_P_19.3_I_8_ (6)

P_4 (G)_ → HP (7)

Sn_24_P_19.3_I_8_ + P_4_ → BP + SnI_2_ (8)

### 3.2. Preparation of BPQDs

**Figure 2:**
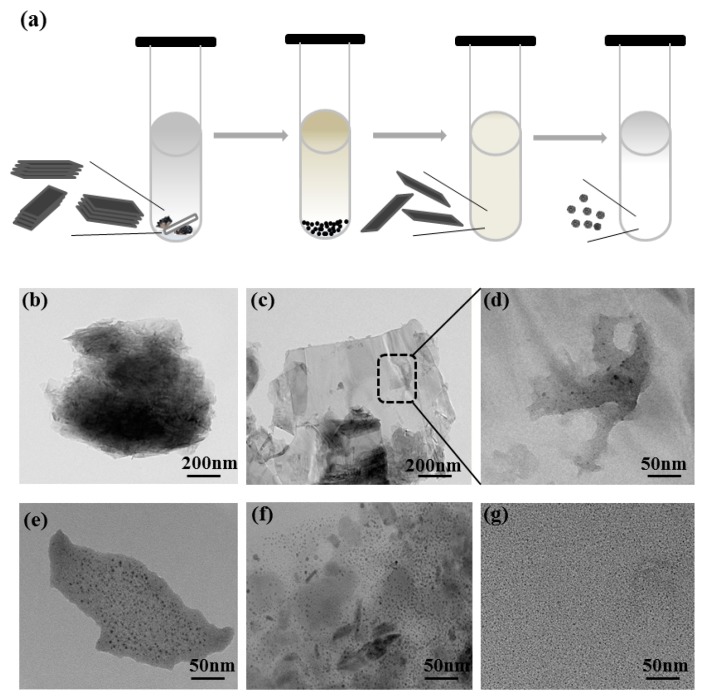
(**a**) Schematic of bulk BP used to synthesize black phosphorus quantum dots (BPQDs) in ethanol using a microwave (MW)-assisted technique. TEM images of intermediate BPQDs at different disintegrating times: (**b**) MW-1 at 11 min, (**c**) MW-2 at 5 min, (**d**) magnified image from (c), (**e**) MW-2 at 10 min, (**f**) MW-2 at 20 min, and (**g**) MW-2 at 30 min.

BPQDs were fabricated by employing a BP single crystal as a precursor by MW-assisted LPE technology in ethanol solution, and the corresponding processes are depicted in Figure 2, which involved a two-step microwave process. In detail, the first microwaving step (termed “MW-1”) weakened the van der Waals (vdW) attractive interactions between the adjacent BP layers. In this step, the ethanol molecule intercalated into the layers of the BP under microwave-energy stimuli at 70 °C. Following this line of thought, the presence of ethanol is crucial for the exfoliation of the BP single crystal into a lower-dimensional structure. In the second microwaving step (called “MW-2”), the intercalated solvent vaporized after receiving heat from the BP heated at 80 °C, and this vapor exerted a bursting pressure greater than the pressure that exists due to the inter-layer vdW attractive interactions. Meanwhile, under the action of the rotor shear force in the equipment, the vdW attractive interactions were further reduced and formed the layered structure (2D BP sheet) or even a quantum dot [10,29,30]. Compared to generally used solvents, such as *N*-methyl-2-pyrrolidone (NMP) [31], dimethylformamide (DMF) [32], dimethyl sulfoxide (DMSO) [32], *N*-cyclohexyl-2-pyrrolidone (CHP) [9], and isopropyl alcohol (IPA) [33], ethanol is a cheaper organic solvent with a low boiling point, which makes the utilization of its intrinsic properties in applications more convenient, and it can be removed easily after processing. More importantly, high-quality BPQDs can be obtained within one hour by this proposed method, which helps prevent oxidation and maintains original crystallinity. The mechanism behind the rapid exfoliation can be attributed to the easy vaporization of ethanol, and the vapor produced by this vaporization exerted a bursting pressure on the layers of the BP ensuring their rapid exfoliation. Finally, the top 50% of the supernatant could be collected for further analysis.

Although it is clear that the ethanol molecule plays an important role during the formation of BPQDs, in-depth understanding of the conversion processes is also valuable for the development of fabrication technology. By monitoring the morphology evolution at a selected disintegration time, the particle size was seen to gradually reduce as the interaction time increased. The dispersion was produced by exfoliating the bulk BP single crystal for the same exfoliation time (11 min) in MW-1, followed by different times (5, 10, 20, and 30 min) in MW-2. The TEM images in Figure 2b show that the solvent was inserted into the black phosphorus layers to weaken its van der Waals attractive interactions in the MW-1 stage. When the processing time was further prolonged at the MW-2 stage (Figure 2c), BP sheet fragmentation occurred and BPQDs were formed on the BP sheet surface (see Figure 2d). When the processing time increased from 10 to 30 min, as seen in Figure 2e–g, the BP sheet was gradually reduced and then completely converted into BPQDs. 

**Figure 3:**
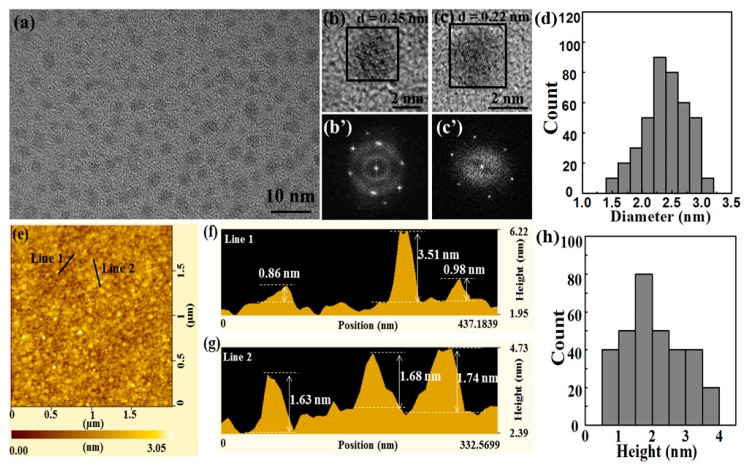
Structural characterization of the BPQDs in ethanol: (**a**) TEM of the BPQDs, (**b**,**c**) HRTEM and (**b’**,**c’**) the corresponding FFT of the BPQDs, (**d**) histogram of the lateral size measured, (**e**) AFM image of the BPQDs, (**f**,**g**) statistical analysis of the heights of the BPQDs measured by AFM, and (**h**) histogram of the height measured.

In order to explore the detailed crystal structure properties of the prepared BPQDs, TEM analysis and atomic force microscopy (AFM) were carried out. The TEM image of BPQDs fabricated by the MW-assisted LPE technology is shown in Figure 3a. It can be seen that the monodispersed BPQDs had an average diameter of 2.5 nm according to the size distribution inserted in Figure 3d, suggesting the feasibility of preparing BPQDs using the MW-assisted method. The HRTEM images of the prepared BPQDs (Figure 3b,c) indicated that the BPQDs were well crystallized [12], further confirming the high quality of BP crystal preparation using the scalable mineralization method. The lattice fringes derived from the HRTEM image and the corresponding fast Fourier transform pattern (Figure 3b’,c’) were highly consistent with the values of 0.25 and 0.22 nm from different BPQDs. The AFM images of the BPQDs, as given in Figure 3e–g, demonstrated the topographic morphology of BPQDs. This agreed with the TEM images, and the heights of the peaks were between 0.86 and 3.51 nm, corresponding to BPQDs of between 1 and 4 layers. The statistical AFM analysis, as given in Figure 3h, revealed the average thickness of BPQDs to be equal to 2.19 ± 1.33 nm.

**Figure 4:**
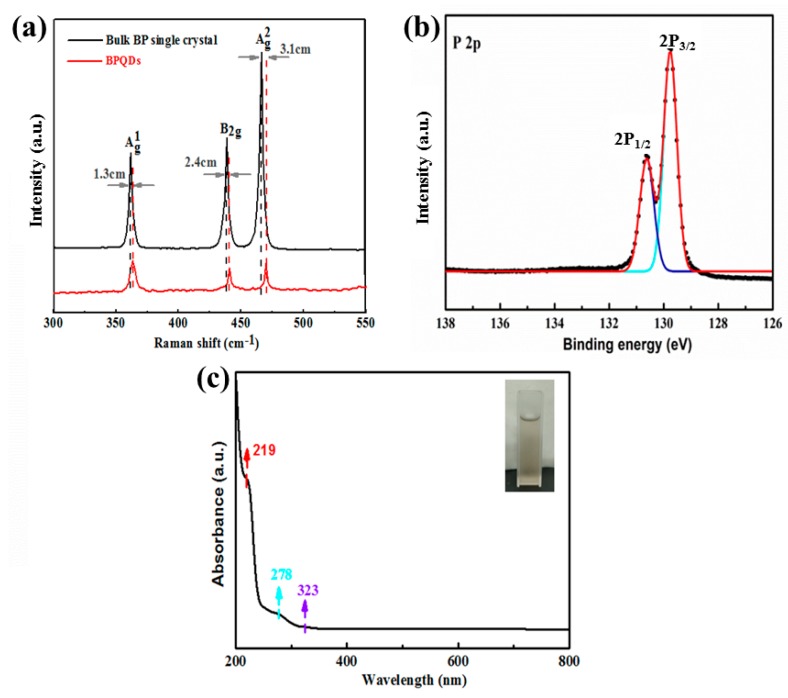
(**a**) Raman spectra of bulk BP and BPQDs, (**b**) high-resolution XPS spectrum of P 2p of the BPQDs, and (**c**) UV–vis–NIR absorption spectrum of the BPQDs. Inset: image of the BPQDs’ dispersion.

Raman spectroscopy was carried out to explore the structural transformation of bulk BP single crystals and BPQDs. As shown in Figure 4a, three main characteristic peaks, centered at approximately 361.6, 438.1, and 466.5 cm^−1^, were noticeable, which correspond to the vibrations of $A_{g}^{1}$, $B_{2g}$, and $A_{g}^{2}$ phonon modes, respectively [11,12,18]. In the case of $B_{2g}$ and $A_{g}^{2}$ modes, the P atoms oscillate in the layer plane, while the P atoms vibrate out of the plane in the case of the $A_{g}^{1}$ phonon mode [34]. In addition, the similar three characteristic peaks again indicate that the BPQDs exfoliated from bulk BP single crystals retain an initial crystalline nature. It is worth noting that, compared to bulk BP, the characteristic peak intensity of all three modes, namely $A_{g}^{1}$, $B_{2g}$, and $A_{g}^{2}$, of the BPQDs is significantly weakened, which can be attributed to the reduction in thickness and lateral dimension [11,12,18]. Moreover, all three modes of BPQDs are redshifted about 1.3, 2.4, and 3.1 cm^−1^, respectively, indicating the existence of few layers [35,36], which is consistent with our TEM and AFM analysis results. It is a fact that BP is sensitive to the atmosphere (including oxygen and water) and can be easily oxidized to phosphoric acid or P_2_O_5_ [37], degrading its intrinsic photoelectric properties. Therefore, we further analyzed the valence of the P element by using X-ray photoelectron spectroscopy (XPS), as shown in Figure 4b. The high-resolution spectrum of the P 2p peak shows that there were only the two 2p_3/2_ and 2p_1/2_ components at 129.7 and 130.6 eV, respectively, indicating the high quality of the prepared BPQDs [32,37]. Notably, the oxidized phosphorus (i.e., PO*_x_*) sub-band was not observed, showing the considerable stability of the prepared BPQDs, which can not only be attributed to the BP single crystal as a raw material of high quality but also to the short experimental time. Due to the great air tolerance of BP single crystals, MW-assisted technology, applied to BP single crystals, is believed to be a promising strategy to produce high-performance BPQDs for future application. Ultraviolet (UV)–visible (vis)–near infrared (NIR) absorption spectroscopy was performed to explore the absorption characteristics of BPQDs. As shown in Figure 4c, this exhibited three characteristic absorption peaks located at 219, 278, and 323 nm. On the contrary, the optical absorbance beyond 400 nm was relatively low, which is consistent with recent reports [38]. It should be noted that the thickness and size distribution of BPQDs are highly dependent on the organic solvent, resulting in the different absorbance peaks of BPQDs. By regulating the organic solvent, the absorption spectrum can cover a wide range from UV to NIR, revealing the potential for optoelectronic applications [9].

## 4. Conclusions

In summary, we synthesized high-quality BP single crystals by using a scalable mineralization method. Using BP single crystals as starting materials, high-quality BPQDs can be quickly and efficiently obtained by exfoliating the BP single crystals in ethanol using MW-assisted LPE technology. During this transformation process, ethanol, as an organic solvent, can efficiently produce ultra-small black phosphorus quantum dots at lower temperatures, making the utilization of BPQDs’ intrinsic properties in applications more convenient. A short experimental time and gentle experimental conditions can effectively prevent oxidation and maintain the original crystallinity. Furthermore, the prepared BPQDs showed extreme stability in air, suggesting that our method has considerable potential for further application. Based on experimental observations, a layer-by-layer disintegration process was proposed to explain the generation of BPQDs during the MW-assisted exfoliation method. We anticipate that this discovery will prove extremely helpful in promoting the development of efficient and fast synthesis for the high-quality nanofabrication of BPQDs.
